# Supplementary material for: Plant-Pathogenic Ralstonia Phylotypes Evolved Divergent Respiratory Strategies and Behaviors To Thrive in Xylem
Source: mBio. 2023 Feb 6;14(1):e03188-22. doi: 10.1128/mbio.03188-22 (PMC9973335; doi:10.1128/mbio.03188-22)
Supplement: FIG S1 [file mbio.03188-22-s0002.pdf]

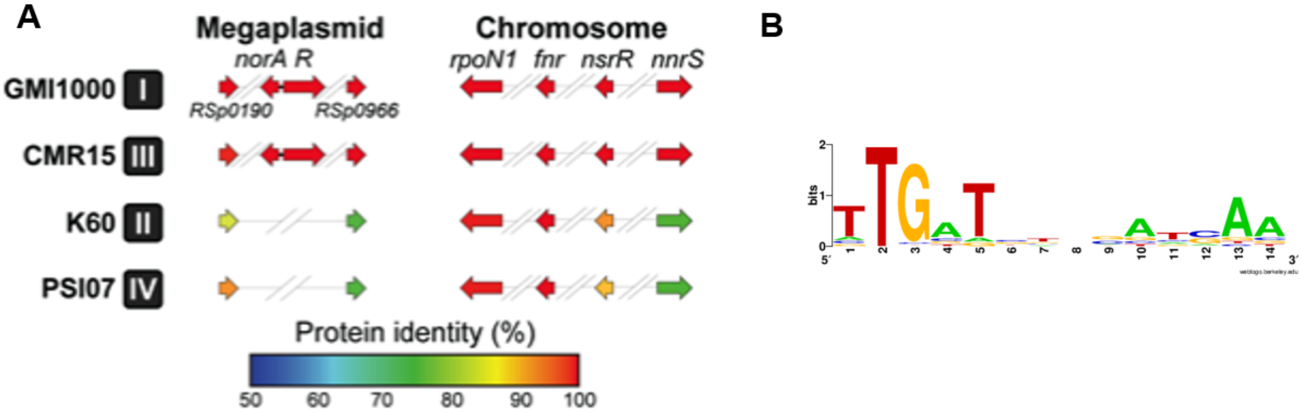

C

Gene	Locus tag in GMI1000	Gene Function	GMI1000 FNR Site?	GMI1000 Distance	GMI1000 Binding sequence	CMR15 FNR Site?	CMR15 Distance	CMR15 Binding sequence	PSI07 FNR Site?	PSI07 Distance	PSI07 Binding sequence
narK1	RSp0972	Nitrate importer	Yes	-200	TTGACGTGAATTAA	Yes	-209	TTGACATGAATTAA	Yes	-177	TTGACCGCTGTCAA
narG	RSp0974	Membrane bound nitrate reductase	Yes	-458	ATGATCCCGATCAT	Yes	-458	ATGATCCCGATCAT	Yes	-247	TTGATTCCAACCAG
nosZ	RSp1368	Nitrous oxide reductase	No			No			NA		
norA	RSp0958	Binds nitric oxide	Yes	-86	TTGATTAAAGGAAA	Yes	-86	TTGATTAAAGGAAA	NA		
norR	RSp0959	NorR family transcriptional regulator	May share with *norA*			May share with *norA*			NA		
aniA	RSp1503	Nitrite reductase	Yes	-156	TTGATGTATATCAG	Yes	-194	TTGATGTATATCAG	Yes	-223	TTGATCAACTCCAA
norB	RSp1505	Nitric oxide reductase	Yes and may share with *norB*	-96	CTGATATACATCAA	Yes and may share with *norB*	-96	CTGATATACATCAA	Yes	-201	TTGGAGTTGATCAA

Figure S1. Denitrification-related regulators and FNR predicted binding sites in genomes of phylotype representative strains in the RSSC. A) The presence or absence and % amino acid identity of all denitrification-associated regulators were determined, using the strain GMI1000 genome for comparison. The *norAR* gene pair is predicted to be nitric oxide-responsive, while *rpoN1* controls gene expression in response to nitrogen starvation and *nsrR* responds to nitrite/nitric oxide. NnrS in other systems responds to nitric oxide. The majority of the regulators, particularly FNR-like RSp0190, RSp0966 and *fnr* (RSc1283), are predicted to be oxygen-responsive. **B)** Predicted binding site consensus (logo) for the RSSC FNR-like regulon. **C)** Predicted FNR-like regulator binding sites located upstream of start site for inorganic nitrogen metabolism genes in strains GMI1000, CMR15, and PSI07.
