# Supplementary material for: Plant-Pathogenic Ralstonia Phylotypes Evolved Divergent Respiratory Strategies and Behaviors To Thrive in Xylem
Source: mBio. 2023 Feb 6;14(1):e03188-22. doi: 10.1128/mbio.03188-22 (PMC9973335; doi:10.1128/mbio.03188-22)
Supplement: FIG S2 [file mbio.03188-22-s0003.pdf]

Figure S2 (Legend below)

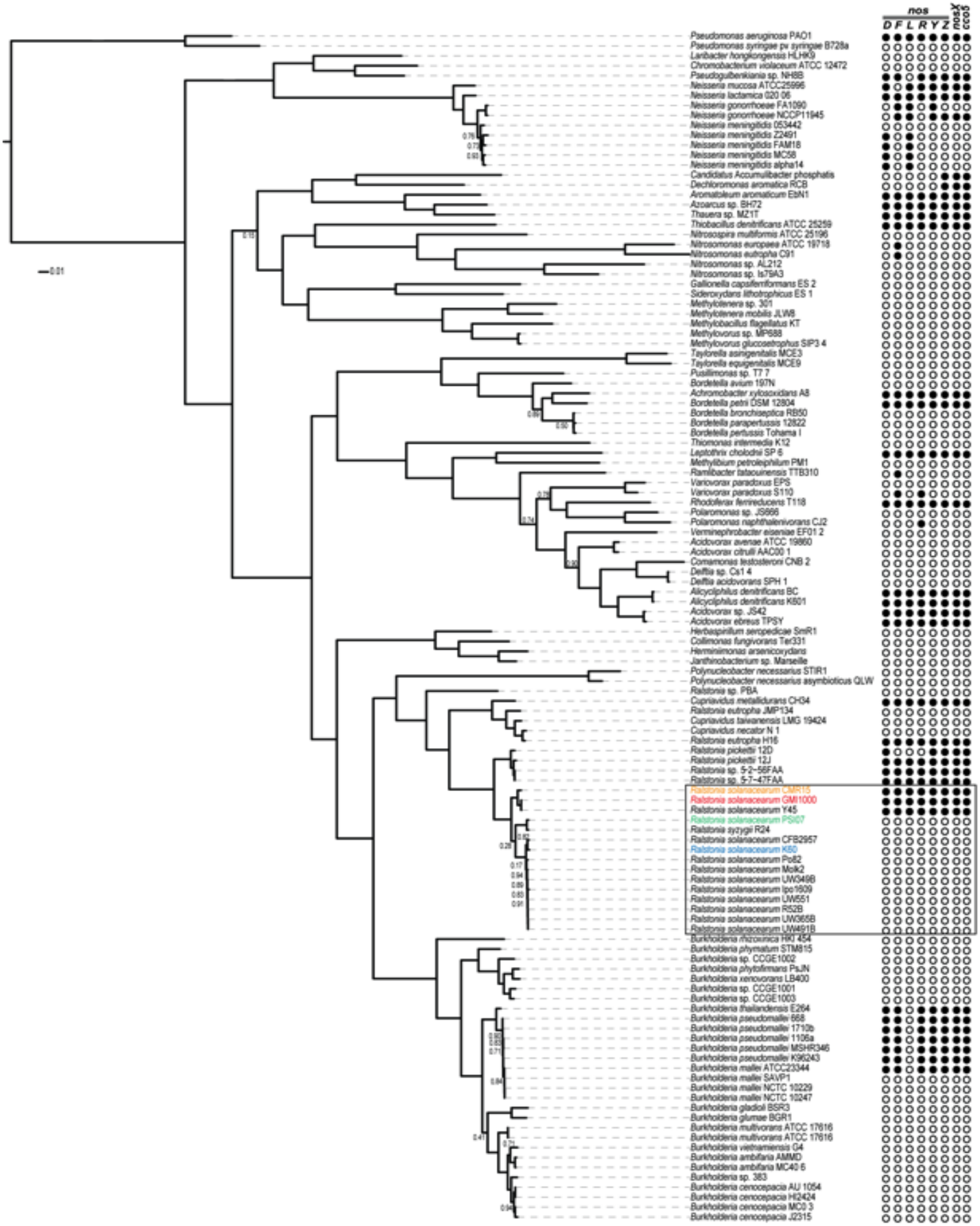

Figure S2. The *nos* cluster, containing the genes required for the last step of denitrification, has been repeatedly laterally transferred across the Betaproteobacteria. All complete Betaproteobacteria genomes from NCBI available in 2012 were compared via MLST using 31 loci as described (22). The corresponding sequences were extracted, corrected, aligned and concatenated using the ORFcor pipeline developed by Klassen and Currie (23) and MUSCLE 3.7 was used for alignment. The tree was constructed using Fasttree v2.1.3 and bootstrap values are found at the nodes. Any values above 0.95 were removed for simplicity. Nos protein sequences were identified using a strict threshold for annotation (>40% identity over 70% of the query sequence length for all but NosF, which had a cut-off of 30% because it is more diverse). *P. stutzeri* CAA37 Nos sequences were used as the query sequences using blastp in the BLAST+ package. Circles represent the 6 standard components of the nitrous oxide reductase gene cluster (listed in alphabetical order from *nosD* to *nosZ*) and 2 additional genes (*nosX* and *cco5*) that often co-segregate. White circles indicate absence of a gene and black circles indicate its presence. All strains in the RSSC are framed in the black rectangle. Strains chosen to represent each of the four phylotypes are in color: phylotype I representative, GMI1000, is in red; phylotype II K60 in blue; phylotype III CMR15 in orange; phylotype IV PSI07 in green.
