# Supplementary material for: Plant-Pathogenic Ralstonia Phylotypes Evolved Divergent Respiratory Strategies and Behaviors To Thrive in Xylem
Source: mBio. 2023 Feb 6;14(1):e03188-22. doi: 10.1128/mbio.03188-22 (PMC9973335; doi:10.1128/mbio.03188-22)
Supplement: FIG S3 [file mbio.03188-22-s0004.pdf]

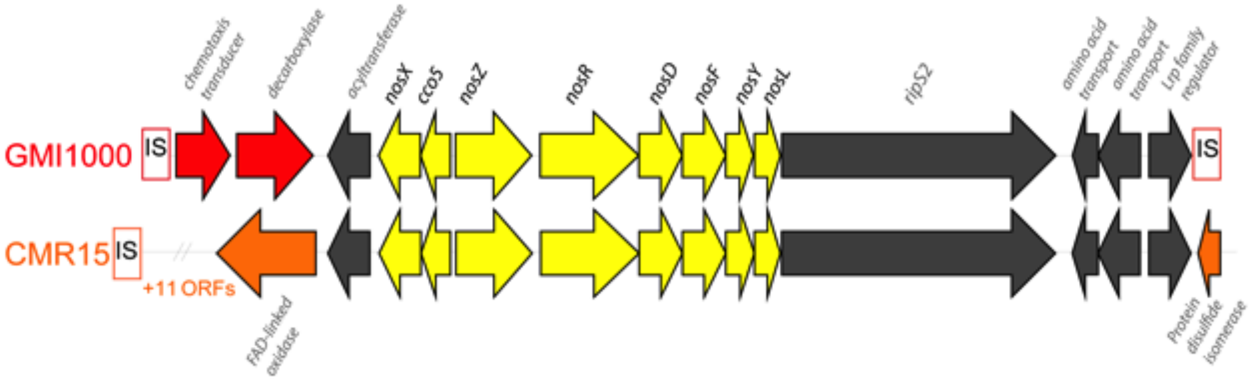

Figure S3. *R. solanacearum* strains in phylotypes I and III share a highly conserved nitrous oxide reductase gene cluster (in yellow). All genes near the *nosZ* ORF that lie between two predicted insertion sequences in phyl. I strain GMI1000 genome (locus tags RSp1362-RSp1378) are aligned to the region in phyl. III strain CMR15 (CMR15_mp30001-CMR15_mp30026). Genes found only in GMI1000 are in red while genes in orange are specific to CMR15. Predicted functions based on NCBI and Phyre2 protein structure modeling are listed in gray italics near each ORF lacking an annotation.
