# Supplementary material for: Plant-Pathogenic Ralstonia Phylotypes Evolved Divergent Respiratory Strategies and Behaviors To Thrive in Xylem
Source: mBio. 2023 Feb 6;14(1):e03188-22. doi: 10.1128/mbio.03188-22 (PMC9973335; doi:10.1128/mbio.03188-22)
Supplement: FIG S4 [file mbio.03188-22-s0005.pdf]

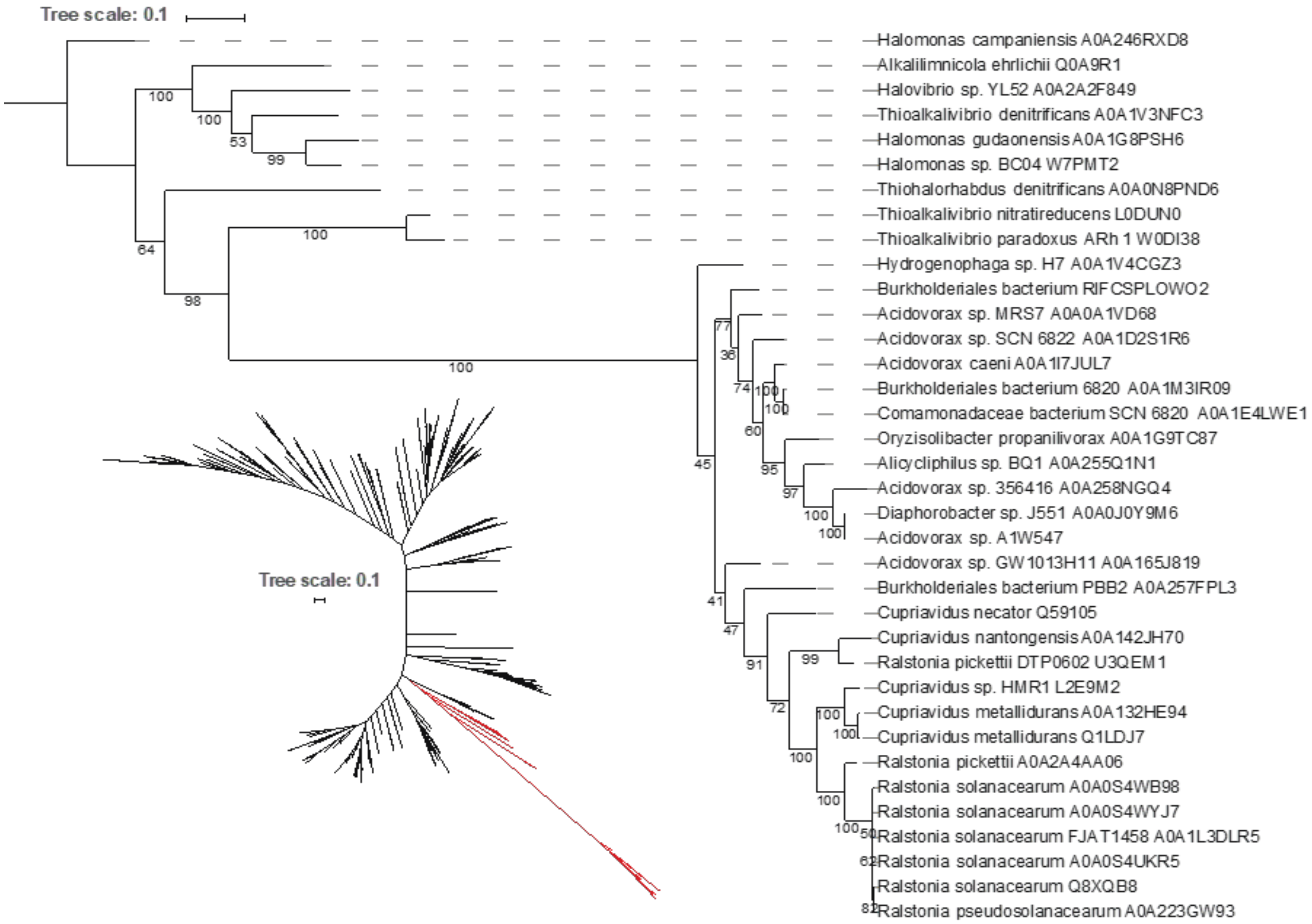

Figure S4. NosZ protein phylogeny. Inset: unrooted tree showing full phylogeny of all NosZ protein sequences from uniprot. The clade containing *Ralstonia* is highlighted in red. The clade containing Uniprot proteins from the RSSC is shown in detail; they form a monophyletic clade of highly related sequences.
