# Supplementary material for: Plant-Pathogenic Ralstonia Phylotypes Evolved Divergent Respiratory Strategies and Behaviors To Thrive in Xylem
Source: mBio. 2023 Feb 6;14(1):e03188-22. doi: 10.1128/mbio.03188-22 (PMC9973335; doi:10.1128/mbio.03188-22)
Supplement: FIG S6 [file mbio.03188-22-s0007.pdf]

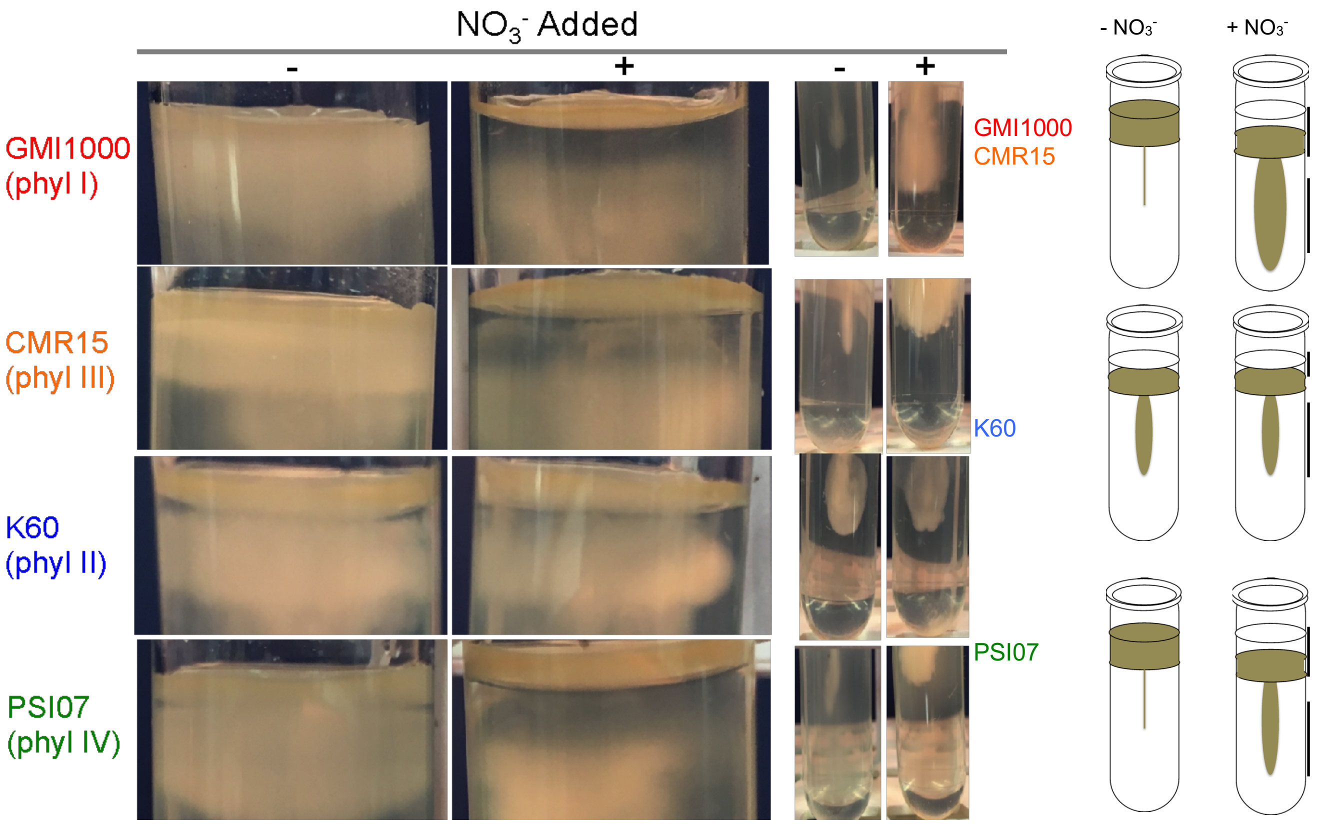

Figure S6. Oxygen preference assessments of *R. solanacearum* phylotype representatives. CPG overnight cultures of each strain were pelleted and re-suspended in water to an O.D.$_{600}$ of 1.0. 10uL of these cell suspensions were stab inoculated and slowly released by a pipette into a plastic tube filled with 20 mL 0.2% semisolid VDM agar with or without nitrate added. Following one week of 28°C incubation without shaking, tubes were imaged. To the right, diagrams depict the general trends.
