# Supplementary material for: Plant-Pathogenic Ralstonia Phylotypes Evolved Divergent Respiratory Strategies and Behaviors To Thrive in Xylem
Source: mBio. 2023 Feb 6;14(1):e03188-22. doi: 10.1128/mbio.03188-22 (PMC9973335; doi:10.1128/mbio.03188-22)
Supplement: FIG S7 [file mbio.03188-22-s0008.pdf]

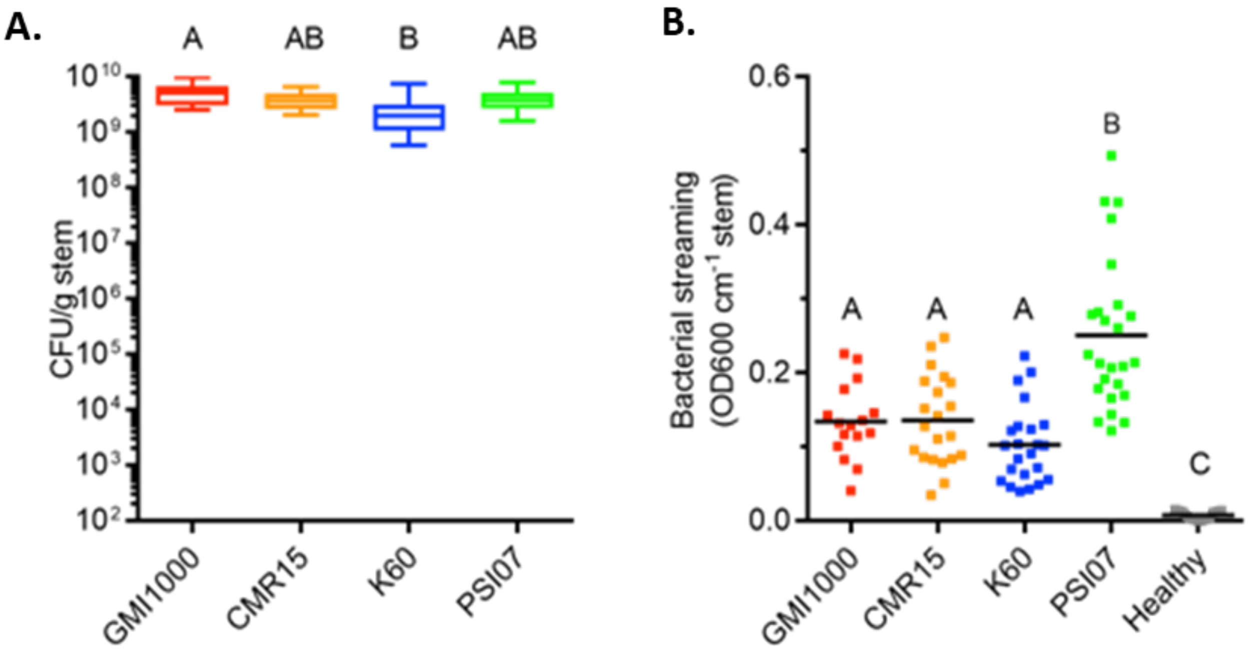

Figure S7. Complete and incomplete RSSC denitrifiers adhere to xylem differently. Representative tomato-colonizing strains (GMI1000, K60, CMR15, PSI07) were inoculated into tomato stem via cut petiole inoculation (1×10^3 CFU). At the first sign of wilting symptoms, **(A)** total bacterial populations in the stem were enumerated by dilution plating or **(B)** bacterial attachment to stem xylem was assessed by measuring planktonic cells that floated or swam out of cut stem tissue. One cm of stem was incubated in water with 85 rpm shaking. After 90 min, bacterial density in the water was measured by $OD_{600\ nm}$. Letters indicate $P<0.05$ by ANOVA with Tukey's multiple comparison test; (A) N=14-15 plants and (B) N=16-25 plants per condition.
