# Supplementary material for: Plant-Pathogenic Ralstonia Phylotypes Evolved Divergent Respiratory Strategies and Behaviors To Thrive in Xylem
Source: mBio. 2023 Feb 6;14(1):e03188-22. doi: 10.1128/mbio.03188-22 (PMC9973335; doi:10.1128/mbio.03188-22)
Supplement: TABLE S1 [file mbio.03188-22-s0001.pdf]

Table S1. *Ralstonia* spp. strains used in this study.

Strain	Phyl	Host	Origin	Accession Number
**GMI1000**	**I**	**Tomato**	**Guyana**	**Genbank: NC_003295, NC_003296**
Y45	I	Tobacco	China	GenBank: AFWL00000000
F504, FQY_4	I	Tobacco	China	GenBank: NC_020799.1, 021745.1
YC45	I	Ginger	China	GenBank: CP011997.1, CP011998.1
Rs09-161	I	Eggplant	India	GenBank: CM002757.1, CM002758.1
Rs10-244	I	Pepper	India	GenBank: CM002755.1, CM002756.1
R292	I	Mulberry	China	NA
PSS81	I	Tomato	Taiwan	NA
**K60**	**II**	**Tomato**	**United States**	**GenBank: NCTK00000000**
B50	II	Banana	Brazil	EMBL: PRJEB7421
IBSBF1900	II	Banana	Brazil	EMBL: PRJEB8309
CFBP2957	II	Tomato	French West Indies	EMBL: FP885897, FP885907
UW181	II	Plantain	Venezuela	EMBL: PRJEB8309
Grenada9-1	II	Banana	Grenada	EMBL: PRJEB7428
Po82	II	Potato	Mexico	GenBank: CP002819, CP002820
UW179	II	Banana	Colombia	EMBL: PRJEB7426
UW163	II	Plantain	Peru	EMBL: PRJEB7430
IBSBF1503	II	Cucumber	Brazil	EMBL: PRJEB7433
CFBP6783	II	Heliconia	French West Indies	EMBL: PRJEB7432
CFBP7014	II	Anthurium	Trinidad	EMBL: PRJEB8309
CFBP1416	II	Plantain	Costa Rica	EMBL: PRJEB7434
CIP417	II	Banana	Philippines	EMBL: PRJEB7437
MolK2	II	Banana	Phillipines	GenBank: CAHW01000040
CFBP3858	II	Potato	Netherlands	EMBL: PRJEB8309
UW349	II	Potato	Brazil	GenBank: JQOI00000000.1
UW365	II	Potato	China	GenBank: JQSI00000000.1
IPO1609	II	Potato	Netherlands	GenBank: CU914168, CUP914166
UW551	II	Geranium	Kenya	GenBank: AAKL00000000
UY031	II	NA	Brazil	GenBank: CP012687.1, CP012688.1
UW491	II	Potato	Colombia	GenBank: JQSH00000000.1
RS2	II	Potato	NA	EMBL: PRJEB8309
**CMR15**	**III**	**Tomato**	**Cameroon**	**EMBL: FP885895, FP885896**
J25	III	Tomato	Kenya	NA
CFBP3059	III	Eggplant	Burkinia Faso	NA
**PSI07**	**IV**	**Tomato**	**Indonesia**	**EMBL: FP885906, FP885891**
R229	IV	Banana	Indonesia	EMBL: FP854059 to FR854085
KACC10722	IV	NA	South Korea	GenBank: CP014702.1, CP014703.1
R24	IV	Clove	Indonesia	EMBL: FR854086 to FR854092
CFBP6727	IV	Potato	Indonesia	NA
MAFF301558	IV	Potato	Japan	NA

NA = not available
