# Supplementary material for: Plant-Pathogenic Ralstonia Phylotypes Evolved Divergent Respiratory Strategies and Behaviors To Thrive in Xylem
Source: mBio. 2023 Feb 6;14(1):e03188-22. doi: 10.1128/mbio.03188-22 (PMC9973335; doi:10.1128/mbio.03188-22)
Supplement: FIG S5 [file mbio.03188-22-s0006.pdf]

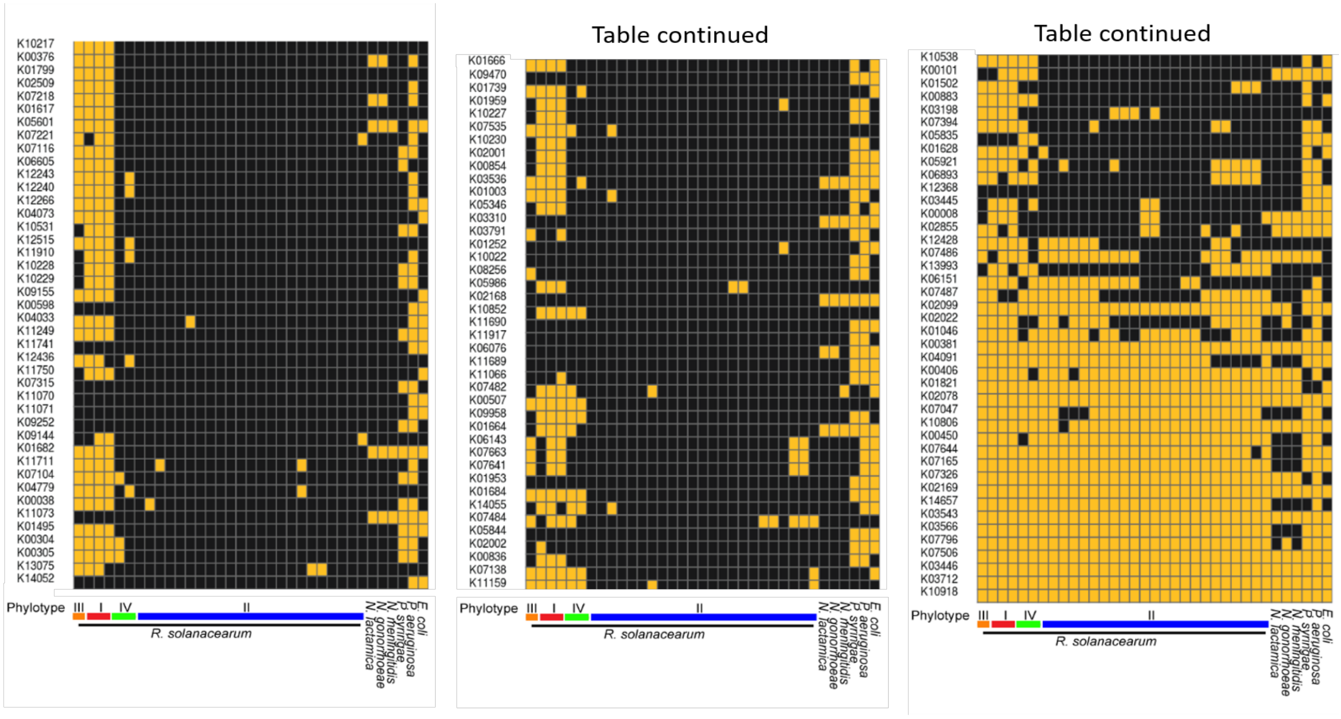

Figure S5. Over-represented KEGG groups in RSSC phylotype I and III strains. HMMer models for KEGG were used to identify the over-represented KEGG families in *R. solanacearum.* Complete denitrifiers I and III shown in red and orange respectively, while incomplete denitrifiers II and IV are shown in blue and green respectively. Presence/absence of overrepresented KEGG families depicted above as yellow or black respectively. Distantly related bacteria with and without complete denitrification were used for comparison (right side of figure).
